# Developing and validating the CE-MACE model to predict 1-year major adverse cardiovascular events post-COPD exacerbation using routine healthcare data

**DOI:** 10.1183/13993003.02555-2025

**Published:** 2026-07-23

**Authors:** Ye Wang, Anne E. Ioannides, Constantinos Kallis, Jennifer K. Quint

**Affiliations:** 1 School of Population Medicine and Public Health, Chinese Academy of Medical Sciences and Peking Union Medical College, Beijing, China; 2 School of Public Health, Imperial College London, London, UK

## Abstract

**Background:**

Patients with COPD are at an elevated risk of cardiovascular events, particularly following acute exacerbations. Existing prediction models underestimate their risk. We developed and validated the CE-MACE model to predict 1-year major adverse cardiovascular event (MACE) risk following a COPD exacerbation.

**Methods:**

Using electronic health records from the UK Clinical Practice Research Datalink Aurum database, we included patients ≥40 years old with moderate or severe COPD exacerbations. The outcome was fatal or non-fatal MACE (acute coronary syndrome, arrhythmia, heart failure or ischaemic stroke). Cause-specific hazard models were used to estimate the coefficient of predictors. Model performance was assessed by Nagelkerke's R^2^, Harrell's C statistic, calibration-in-the-large, calibration slope and decision curve analysis. Internal–external cross validation was used to evaluate model performance across nine geographic regions. A risk score was derived from the original coefficients.

**Results:**

A total of 338 981 patients were included. The overall 1-year cumulative incidence rate of MACE following COPD exacerbation was 5.04% (95% confidence interval 4.96–5.12%). Six predictors were retained: age, exacerbation severity, MACE history, modified Medical Research Council dyspnoea scale, hypertension and diabetes. In internal–external cross validation, the pooled estimation for Nagelkerke's R^2^ was 3.120% (2.945–3.295%) and Harrell's C statistic was 0.752 (0.746–0.759). Calibration-in-the-large was −0.006 (−0.008– −0.004) and the calibration slope was equal to 0.999 (0.977–1.020). These results suggest that the model has clinical utility, with higher net benefit than default strategies across the risk thresholds range from 2% to 18%. Model-driven risk stratification demonstrated significantly different cumulative incidence rates among these risk categories. Sensitivity analysis revealed that the CE-MACE model is generalisable to those patients with any history of COPD exacerbation.

**Conclusion:**

The CE-MACE model highlights the burden of cardiovascular multimorbidity in patients with COPD. The model could help precisely identify those at high risk in clinical practice and promote integrated multidisciplinary healthcare for these patients.

## Shareable abstract

**The CE-MACE model was developed and validated to predict 1-year MACE risk following a COPD exacerbation using routine healthcare data. The model identifies high-risk patients more precisely, providing an opportunity to improve care for people with COPD.** https://bit.ly/4aB9496

## Graphical abstract

**Figure:**
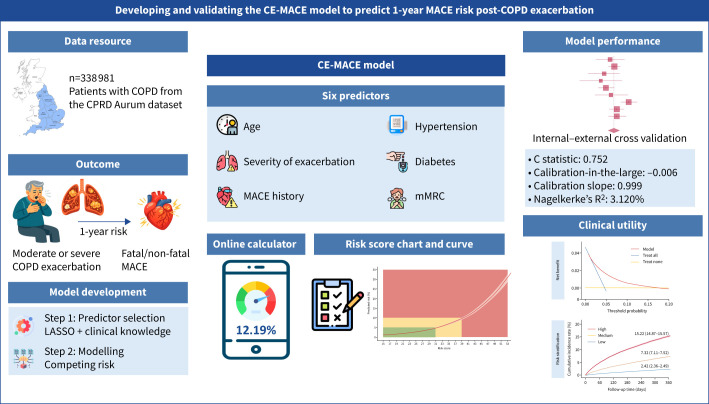
Summary of the study design, data sources, model development, validation strategy and intended clinical application of the CE-MACE model. CPRD: Clinical Practice Research Datalink; MACE: major adverse cardiovascular event; mMRC: modified Medical Research Council dyspnoea score.

## Background

COPD is a common respiratory condition, estimated to affect 10.6% of people 40 years and older in 2020 worldwide, accounting for approximately 480 million cases [1]. People with COPD often have acute episodes of deterioration beyond normal day-to-day variation, resulting in worsening symptoms of breathlessness, sputum volume and cough, which are called acute exacerbations [2]. Patients who have exacerbations face higher risks of death and other adverse outcomes (such as worse quality of life or heart attacks) than those without an exacerbation [3].

Cardiovascular disease (CVD) is a common comorbidity in patients with COPD, affecting between 28% and 70% of patients [4]. Patients with COPD are at increased risk of CVD, and concomitant disease leads to reduced quality of life, increased hospitalisations and worse survival [5]. Significantly increased risks of cardiovascular events (*e.g.* heart failure, heart attacks and irregular heart rhythms) after exacerbation have been previously observed [6–9]. Existing evidence suggests that current CVD prediction models for the general population underestimate the actual risk in people with COPD [10]. Although factors related to increased risk of CVD among patients with COPD have been investigated, precisely identifying those at high risk in clinical practice remains challenging [11, 12].

We have used routinely collected healthcare data to develop and validate a prediction model for use in clinical practice to identify patients at risk of major adverse cardiovascular events (MACE) in the 1-year period after a COPD exacerbation.

## Methods

### Data source

Data from patients with a diagnosis of COPD were extracted from the Clinical Practice Research Datalink (CPRD) Aurum database (December 2024 build). CPRD data are electronic healthcare records routinely collected from general practitioners (GPs) in the UK. The data cover approximately 24% of the UK population and are representative of age, sex and region [13]. Linked secondary care data from Hospital Episode Statistics (HES) and mortality data from the Office for National Statistics (ONS) were also obtained from CPRD [14].

### Study population and study design

The study included healthcare records from CPRD between 1 January 2008 and 31 March 2023. A validated methodology was used to determine COPD diagnosis (codelist: https://github.com/NHLI-Respiratory-Epi/CE-MACE-prediction-model) [15]. The specific diagnostic codes can accurately capture patients with COPD from UK primary care records with a positive predictive value of 86.5%.

We included patients with moderate (managed in primary care) and severe (managed in secondary care) COPD exacerbations from the CPRD data. Whilst the analysis was retrospective, data collection occurred prospectively. Validated algorithms for determining COPD exacerbations were used [16, 17]. A moderate exacerbation was defined as: 1) a medical diagnosis of lower respiratory tract infection or COPD exacerbation, 2) a prescription of COPD-specific antibiotic combined with oral corticosteroids (OCS) for 5–14 days, or 3) a record of two or more respiratory symptoms of COPD exacerbation along with a prescription of COPD-specific antibiotics and/or OCS on the same day [16]. A severe exacerbation (hospitalisation for COPD exacerbation in HES) was defined using International Classification of Diseases, tenth revision (ICD-10) codes and diagnostic positions in the first episode of care described in supplementary table S1 [17]. Exacerbations were considered to be the same event if recorded within 14 days, in which case the highest degree of severity was chosen.

Patients were eligible to be included in the cohort if they: 1) had a diagnostic code for COPD using a validated definition [15], 2) had at least one moderate or severe exacerbation of COPD during the study period, 3) were ≥40 years old, and 4) had at least 1 year of GP practice registration prior to the index date. The index date was the date of the first exacerbation after meeting the latest eligibility criteria. To minimise the effect of complex conditions (especially on respiratory disease) during the COVID-19 pandemic, patients with an index date between 1 January 2020 and 31 December 2021 were not included in the analysis.

Patients were excluded if they met the following criteria: 1) not eligible for HES and ONS linkage, 2) had a MACE within 2 months prior to the index date, and 3) were without at least 1 day of follow-up (supplementary figure S1). All code lists in the cleaning process can be found at our GitHub repository (https://github.com/NHLI-Respiratory-Epi/CE-MACE-prediction-model).

### Follow-up

The start of the follow-up was the index date defined above. The end of the follow-up was the first of outcome, other-cause death, 1-year event-free follow-up, transferred GP practice or end of the study period, whichever came first (supplementary figure S2).

### Outcomes definition

The outcome of this study was fatal or non-fatal MACE, as recorded in HES or ONS data, which was determined using the ICD-10 code in the primary diagnostic position for hospitalisation or the primary cause of death. The composition of MACE was acute coronary syndrome (including myocardial infarction and unstable angina), arrhythmia, heart failure and ischaemic stroke. This definition of MACE is widely accepted in the literature [18]. All other-cause deaths that occurred before the observed outcome were considered as competing risk [19].

### Sample size

The sample size calculation method for time-to-event outcome was used [20]. From previous work, it was assumed that the overall incidence rate of MACE is 0.088 (8.8 per 100 person-year) [6], and the number of candidate predictors to be assessed was assumed to be 30 with 45 degrees of freedom. The required minimum sample size for model development was calculated as 7873, with 693 events, corresponding to 15.4 events per predictor. The actual sample sizes in the cohorts were larger than this minimum required sample size.

### Candidate predictors

Based on subject matter knowledge (both from experts and peer-reviewed scientific literature) and availability from the dataset, 21 candidate predictors were preselected before any data-driven selection. The candidate predictors included demographic characteristics, COPD-related characteristics and history, cardiovascular disease-related characteristics and MACE history, and comorbidities (supplementary table S2). The most recent recorded values 1 year before or at the time of index date were used for demographic and COPD-related characteristics. Any records of disease history before the index date were used for history of MACE and comorbidities.

### Missing values

The patterns of missing values for all the candidate predictors were checked before modelling. We excluded patients with missing values on smoking status (1.56%) or body mass index (BMI) (7.02%) at little cost to the overall data collection owing to their low proportion of missingness. For Global Initiative for Chronic Obstructive Lung Disease (GOLD) stage and modified Medical Research Council (mMRC) dyspnoea scale, missing indicators were used under an assumption of missing not at random (MNAR), which is recommended as proper practice where data are assumed to be MNAR and missingness is allowed at model deployment [21]. Other variables did not have any missingness in the data. Therefore, the model does not allow any missing values in any variables other than mMRC and GOLD stage at deployment.

### Predictor selection and model specification

To determine the optimal format of continuous variables to be included in model specification, linear, restricted cubic spline and categorical relationships between predictors and outcome were fitted and compared (supplementary figure S3, supplementary table S3). The optimal format was determined by the contributions to the model and the complexity of specific predictors. The format with less complexity and a higher degree of explanation was considered to be the optimal format. The four MACE history variables were combined as one single predictor based on their regression coefficients, leading to a reduction in the degrees of freedom from four to one (supplementary table S4).

Five model specifications were developed and compared. First, a model using six conventional cardiovascular risk factors (age, sex, smoking status, BMI, hypertension and diabetes) was fitted as the reference model (specification 1). Second, the full model including all 18 candidate predictors was fitted (specification 2), followed by an extended model adding two interaction terms (specification 3). Afterwards, the least absolute shrinkage and selection operator (LASSO) procedure was performed with the expectation of selecting important predictors from the full model with penalisation to reduce overfitting, using λ=1 se as a regularisation parameter (specification 4). In specification 5 two additional predictors based on clinical knowledge were manually added to model 4. Finally, based on the models’ performance, complexity and interpretability, a six-predictor model (specification 5) was specified as the final model (supplementary figure S4).

### Statistical analysis

Descriptive characteristics are presented as n (%), mean±sd or median (interquartile range (IQR)), as appropriate.

Cause-specific hazard models that simultaneously account for competing risks were used to estimate coefficients for predictors. The predicted risk was calculated following the formula: *P*_predicted_=1–(1–*R*_0_)^exp(*β*_1_*X_1_+*β*_2_*X_2_+…+*β*_n_*X_n_), in which *R*_0_=1–exp(–H_0_), representing the baseline risk (predicted risk when all predictors are set to 0) derived from the baseline hazard H_0_; *X*_n_ is the predictor; and *β*_n_ is the corresponding coefficient.

### Model performance

To account for the heterogeneity among healthcare services between geographical regions, we used all the available data to develop the model and then used internal–external cross validation to evaluate the model performance instead of partitioning the data into development and validation cohorts [22]. In this process, the hold-out cluster is used to evaluate the performance of the model developed in all other clusters. The final model is fitted using all clusters. In this study, a cluster was each of the nine regions defined in the CPRD dataset (North East, North West, Yorkshire and the Humber, East Midlands, West Midlands, East of England, London, South East, and South West). These steps were repeated taking out a different cluster each time, thereby allowing the generalisability and heterogeneity of performance to be examined across clusters. Random-effects meta-analysis was used to pool the performance estimates for each region. Model performance was evaluated in terms of overall performance, discrimination, calibration and clinical utility. Overall performance was assessed using Nagelkerke's R^2^, indicating the variation explained by the model. Discrimination was assessed by Harrell's C statistic. Calibration metrics included calibration-in-the-large and calibration slope, as well as visual assessment by calibration plot. Decision curve analysis was used to assess the clinical utility. Net benefit was calculated in this analysis to assess the trade-off between the benefits of true positives and the potential harms that may arise from false positives across a range of threshold probabilities, with the “treat all” and “treat none” strategies as references. “Treat all” represents a hypothetical strategy in which all individuals are classified as high risk and would undergo a downstream clinical action (*e.g.* preventive intervention), regardless of their predicted risk. “Treat none” represents the opposite strategy, in which no individual is classified as high risk, and no downstream action is taken. The term “treat” in decision curve analysis refers broadly to risk-based decision-making, not to a specific therapeutic intervention [23]. Owing to the large sample size and low risk of optimism bias, net benefit was calculated without the intervention–external cross validation. So, apparent estimates of net benefit values are shown.

For easier implementation of the CE-MACE model, a score chart was developed based on each predictor's coefficient, with the scores derived by rounded 10×original coefficient.

### Risk stratification

According to clinical relevance, patients were stratified as high-risk if the predicted 1-year MACE risk was ≥10%, and at medium-risk if predicted risk ranged from 5% to <10%.

### Sensitivity analysis

Owing to the study design, most of the patients included had no prior COPD exacerbation history before the index date. To test the generalisability of the CE-MACE model for those patients with any history of exacerbation, which is common in clinical settings, we conducted a sensitivity analysis undertaking internal–external cross validation of the CE-MACE model using subsequent exacerbations as the unit of analysis. In this process, patients were included in the analysis more than once if they experienced multiple subsequent exacerbations separated by >1 year during the study period. Metrics of overall performance, discrimination, calibration and clinical utility were all estimated.

We used Stata version 18.5 (StataCorp LLC, Texas, USA) for data preparation and R version 4.4.3 (R Foundation for Statistical Computing, Vienna, Austria) for analysis. We did not show any p-values because it is easy to have statistical significance under the <0.05 criteria given the large sample size in this study. The Transparent Reporting of a multivariable prediction model for Individual Prognosis Or Diagnosis plus Artificial Intelligence (TRIPOD-AI) guidance was followed during the modelling process and manuscript drafting [24].

### Patient and public involvement

Patients and members of the public were not directly involved in study design, data collection, data analysis, data interpretation or writing of the report.

## Results

### Baseline characteristics of study cohorts

A total of 338 981 patients were included for analysis, among whom 15 802 had an outcome during the 1-year follow-up after a moderate or severe exacerbation. Summary baseline characteristics of the study population stratified by outcome are displayed in table 1. The overall 1-year cumulative incidence rate of MACE was 5.04% (95% confidence interval (CI) 4.96‒5.12%) (supplementary figure S5).

**TABLE 1:** Baseline characteristics of the study population stratified by outcome

Variables	Overall	Patients with outcome	Patients without outcome^#^
**Total participants (n)**	338 981	15 802	323 179
**Male sex**	169 505 (50.00)	9267 (58.64)	160 238 (49.58)
**Mean±sd age (years)**	70.25±11.45	75.77±10.17	69.98±11.44
**Smoking status**			
Current smoker	115 468 (34.06)	4195 (26.55)	111 273 (34.43)
Ex-smoker	209 290 (61.74)	10 854 (68.69)	198 436 (61.40)
Never-smoker	14 223 (4.20)	753 (4.77)	13 470 (4.17)
**Mean±sd BMI (kg·m^−2^)**	27.38±6.54	27.95±6.83	27.35±6.52
**Severity of current exacerbation**			
Moderate	290 293 (85.64)	12 116 (76.67)	278 177 (86.08)
Severe	48 688 (14.36)	3686 (23.33)	45 002 (13.92)
**COPD exacerbation in last** **12 months**			
<2 moderate and no severe	322 509 (95.14)	14 821 (93.79)	307 688 (95.21)
≥2 moderate or ≥1 severe	16 472 (4.86)	981 (6.21)	15 491 (4.79)
**ICS use in last 12** **months**	217 339 (64.12)	10 158 (64.28)	207 181 (64.11)
**GOLD stage (FEV_1_ % pred)**			
1 (≥80)	48 141 (14.20)	1676 (10.61)	46 465 (14.38)
2 (50–<80)	94 812 (27.97)	4048 (25.62)	90 764 (28.08)
3 (30–<50)	45 979 (13.56)	2270 (14.37)	43 709 (13.52)
4 (<30)	13 094 (3.86)	619 (3.92)	12 475 (3.86)
Missing	136 955 (40.40)	7189 (45.49)	129 766 (40.15)
**mMRC dyspnoea scale**			
0	29 176 (8.61)	825 (5.22)	28 351 (8.77)
1	69 961 (20.64)	2491 (15.76)	67 470 (20.88)
2	52 901 (15.61)	2592 (16.40)	50 309 (15.57)
3	29 746 (8.78)	1903 (12.04)	27 843 (8.62)
4	6769 (2.00)	491 (3.11)	6278 (1.94)
Missing	150 428 (44.38)	7500 (47.46)	142 928 (44.23)
**Prior history of MACE**			
ACS	41 223 (12.16)	4463 (28.24)	36 760 (11.37)
Arrhythmia	57 983 (17.11)	6401 (40.51)	51 582 (15.96)
Heart failure	35 352 (10.43)	4924 (31.16)	30 428 (9.42)
Stroke	29 709 (8.76)	2603 (16.47)	27 106 (8.39)
**Statins use in last 12** **months**	158 732 (46.83)	9729 (61.57)	149 003 (46.11)
**Comorbidity**			
Hypertension	173 699 (51.24)	10 382 (65.70)	163 317 (50.53)
Diabetes	61 234 (18.06)	4583 (29.00)	56 651 (17.53)
Asthma	73 868 (21.79)	2984 (18.88)	70 884 (21.93)
Depression	105 142 (31.02)	4268 (27.01)	100 874 (31.21)
Anxiety	66 520 (19.62)	2840 (17.97)	63 680 (19.70)
GORD	73 968 (21.82)	3588 (22.71)	70 380 (21.78)
LRTI	197 702 (58.32)	9009 (57.01)	188 693 (58.39)

Data are presented as n (%), unless otherwise stated. ACS: acute coronary syndrome; BMI: body mass index; FEV_1_ % pred: % predicted forced expiratory volume in 1 s; GOLD: Global Initiative for Chronic Obstructive Lung Disease; GORD: gastro-oesophageal reflux disease; ICS: inhaled corticosteroids; LRTI: lower respiratory tract infection; MACE: major adverse cardiovascular event; mMRC: modified British Medical Research Council. ^#^: including censor and competing risk death.

### Predictors and prediction

The CE-MACE model includes age, severity of current exacerbation, MACE history, mMRC dyspnoea scale, hypertension history and diabetes history as the final predictors. The multivariable coefficient and hazard ratio (HR) of each predictor are displayed in table 2. An online calculator for the CE-MACE model is available at https://ce-mace.shinyapps.io/shiny/. An example of the online tool can be found in supplementary figure S6.

**TABLE 2:** Estimated effect of predictors in CE-MACE model

Predictors	Coefficient	se	HR (95% CI)
**Age (per decade)**	0.302	0.008	1.35 (1.33–1.37)
**Severity of current exacerbation**	0.531	0.019	1.70 (1.64–1.77)
**mMRC dyspnoea scale**			
0 (Reference)			
1	0.107	0.040	1.11 (1.03–1.20)
2	0.272	0.040	1.31 (1.21–1.42)
3	0.421	0.042	1.52 (1.40–1.65)
4	0.526	0.057	1.69 (1.51–1.89)
Missing	0.326	0.037	1.39 (1.29–1.49)
**MACE history (per score)^#^**	0.177	0.002	1.19 (1.19–1.20)
**Hypertension**	0.181	0.018	1.20 (1.16–1.24)
**Diabetes**	0.325	0.018	1.38 (1.34–1.43)

The predicted risk is calculated as: *P*_predicted_=1–(1–*R*_0_)^exp(*β*_1_*X_1_+*β*_2_*X_2_+…+*β*_n_*X_n_); *R*_0_ was estimated to be 0.00223. CI: confidence interval; HR: hazard ratio; MACE: major adverse cardiovascular event; mMRC: modified Medical Research Council. ^#^: MACE history score = acute coronary syndrome*3 + arrhythmia*4 + heart failure*4 + stroke*2.

### Easy-to-use score chart

**FIGURE 1:**
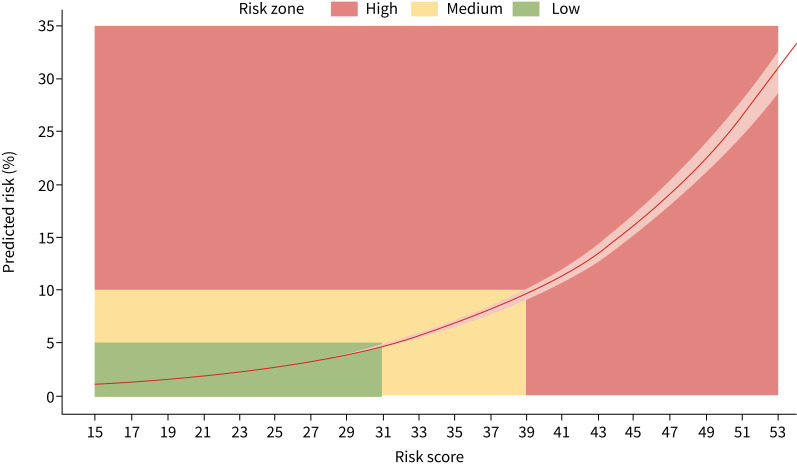
CE-MACE risk scores and the corresponding predicted risk for predicting 1-year major adverse cardiovascular event (MACE) risk post-COPD exacerbation. Risk score in x-axis is the sum score calculated from the chart in table 3. Predicted risk in y-axis means the 1-year predicted risk of fatal or non-fatal MACE (including acute coronary syndrome, arrhythmia, heart failure or stroke). The curve displays the corresponding predicted risk and 95% confidence intervals. Red zone represents high risk (≥10%); yellow zone represents medium risk (5% to <10%); green zone represents low risk (<5%).

The easy-to-use score chart rule was derived from the coefficients of each predictor (table 3), with a corresponding visually predicted risk shown in a curve (figure 1). Around 98% of the scores were between 15 and 54; patients scoring ≥40 were stratified as high risk. The agreement between the predicted risk by risk score and the original model is shown as supplementary figure S7. We prepared an easy-to-use score chart with a two-step tutorial for clinical practice, provided as a separate PDF supplementary document that can be printed and used.

**TABLE 3:** Score chart rule based on rounded coefficients

Terms	Categories	Score	Score assigned
**Age (years)**	40–44	12	
	45–54	15	
	55–64	18	
	65–74	21	
	75–84	24	
	85–94	27	
	95+	30	
**Severity of current COPD**^#^ **exacerbation**	Moderate	0	
	Severe	5	
**MACE history** ^¶^	Acute coronary syndrome	6	
	Arrhythmia	8	
	Heart failure	8	
	Stroke	4	
**Hypertension** ^¶^	No	0	
	Yes	2	
**Diabetes** ^¶^	No	0	
	Yes	3	
**mMRC dyspnoea scale** ^+^	0	0	
	1	1	
	2	3	
	3	4	
	4	5	
	Not available/missing	3	
**Sum score**			

For example, if there is a 70-year-old patient with arrhythmia and heart failure history, and his current COPD exacerbation is severe, then the sum score of this patient is 21+5+8+8=42, corresponding to 12.5% risk of MACE in the next 1 year from figure 4. MACE: major adverse cardiovascular event; mMRC: modified Medical Research Council. ^#^: in the context of the UK healthcare system, a moderate COPD exacerbation is one managed in primary care and a severe COPD exacerbation is one managed in secondary care; ^¶^: any such histories before current exacerbation; ^+^: the last assessment 1 year before current exacerbation.

### Model performance

**FIGURE 2:**
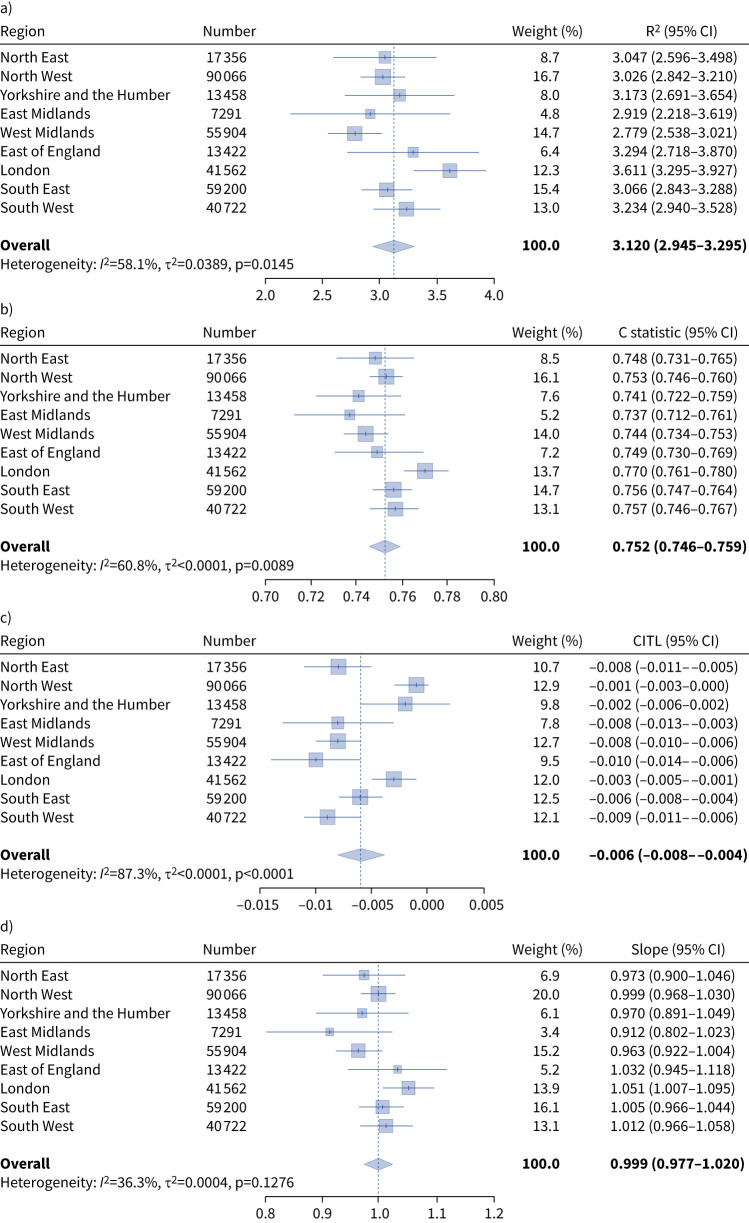
Results from internal–external cross validation of model performance metrics. Plots display region level performance metric estimates and 95% confidence intervals (CIs), and an overall pooled estimate obtained using random-effects meta-analysis. a) Nagelkerke's R^2^; b) Harrell's C statistics; c) calibration-in-the-large; d) calibration slope.

Forest plots of model performance metrics from the internal–external cross validation across regions are displayed in figure 2. Random-effects meta-analysis produced an overall pooled estimate for R^2^ of 3.120% (2.945–3.295%), and a C statistic of 0.752 (0.746–0.759). The overall risk was slightly overestimated, with a meta-analysis pooled estimate for the calibration-in-the-large of −0.006 (−0.008– −0.004). The calibration slope was estimated to be 0.999 (0.977–1.020).

The apparent performance of the CE-MACE model by original format and risk score format is displayed in supplementary figure S8. The internal–external cross validation for the risk score performance metrics is shown in supplementary figure S9.

### Clinical utility

**FIGURE 3:**
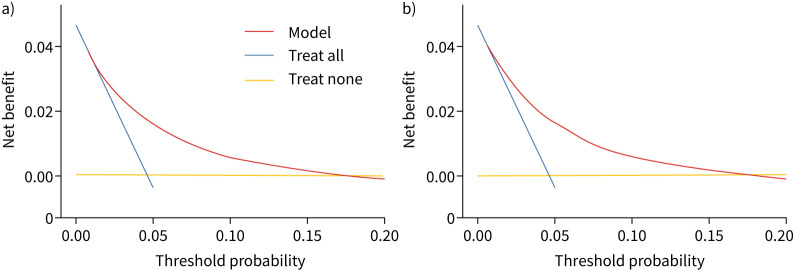
Decision curves analysis of CE-MACE model. a) Model in original format; b) model in risk score format. The net benefit strikes a balance between the benefits (correctly identifying patients who need treatment) and harms (subjecting patients who do not need treatment to side-effects) of using the model. Curves compare the net benefit of using the CE-MACE prediction model with default strategies (“treat all” and “treat none”). The term “treat” in decision curve analysis refers broadly to risk-based decision-making, not to a specific therapeutic intervention.

Decision curve analysis showed that the CE-MACE model had positive net benefit compared to “treat all” and “treat none” strategies across the risk thresholds range from 2% to 18%, indicating clinical utility (figure 3).

### Risk stratification

**FIGURE 4:**
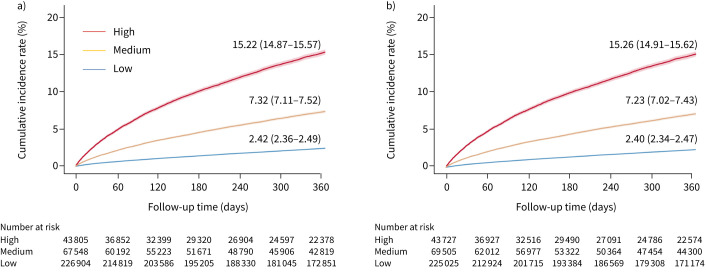
Cumulative incidence rates with 95% confidence intervals of 1-year major adverse cardiovascular event by risk group. a) Model in original format; b) model in risk score format. High-risk group: predicted risk ≥10%; medium-risk group: predicted risk 5% to <10%; low-risk group: predicted risk <5%.

According to the CE-MACE model, patients were divided into low-, medium- and high-risk groups, with cumulative 1-year MACE incidence rates as high as 15% in the high-risk group, 7% in the medium-risk group and 2% in the low-risk group (figure 4).

### Sensitivity analysis

The internal–external cross validation in the sensitivity analysis cohort showed consistent model performance with the main analysis (supplementary figures S10 and S11). The estimate for R^2^ was 2.985% (2.829–3.140%) and for the C statistic was 0.735 (0.731–0.739). The calibration-in-the-large was −0.005 (−0.008– −0.003). The calibration slope was estimated to be 0.920 (0.904–0.936). Decision curve analysis results are shown in supplementary figure S12.

## Discussion

### Principal findings

Using electronic healthcare records in England, we developed and validated the practical and explicable CE-MACE model to predict the 1-year risk of fatal and non-fatal cardiovascular events for patients with COPD following an exacerbation. To our knowledge, this is the first model developed for predicting CVD risk among this at-risk subpopulation.

The CE-MACE model stratifies patients into high, medium and low risk according to the predicted risk. The high-risk group had an average 15% incidence rate of MACE, which was two-fold and seven-fold of those in medium- and low-risk groups, respectively.

### Interpretation of findings

The risk of increased CVD in patients with COPD has been known and discussed for years [25]. The GOLD report proposed that CVD is one of the major multimorbidities with important clinical implications for patients with COPD [2]. The link between COPD and CVD relies on not only the shared exposures and risk factors (*e.g.* ageing, smoking), but also the underlying pathological mechanism, including systemic inflammation [26].

To date, there is no specific guidance or recommendation for the identification and management of cardiovascular events and risk in COPD [27]. Prediction models incorporate risk factor findings into clinical and public health practice through risk assessment, targeted interventions and treatment decision-making. Several prediction models for CVD (*e.g.* Framingham, QRISK3, SCORE2) have been developed in the past two decades [28–30]. However, most previous models were designed for the general population without taking specific high-risk population features into account, leading to suboptimal performance when validated among COPD patients [31]. For example, the QRISK3 score underestimated CVD risk by 52% among patients with COPD [10]. In 2024, QR4 was proposed, which included COPD as one of the new predictors to more accurately estimate CVD risk [32]. The accuracy of this new algorithm among different subpopulations, such as patients with COPD, remains to be validated. Recently, a combination of cardiovascular risk score (CVRS) and coronary artery calcium score (CACS), based on chest computed tomography scans and clinical, functional and laboratory data, was proposed to improve risk stratification of MACEs in patients with COPD [33].

In this study, we did not directly compare the CE-MACE model with previously developed models because of their differences in target population and outcomes. For most previous models, the aim has been to predict 10-year risk of incident CVDs in people who are CVD-free at baseline. Our target population (COPD patients with COPD exacerbations) is different to the general population, with a higher mean age and a large proportion with previous MACE. Among this subpopulation, it is clinically relevant to know who is at short-term risk of a MACE, either incident or recurrent, to guide additional care and integrated management.

The clinical value of our model can be evaluated using a multidimensional set of performance metrics. The variance explained by the model (Nagelkerke's R^2^) was only 3.120%, indicating relatively limited interpretability for individual-level risk prediction. However, in the context of competing risk prediction models, low R^2^ values are methodologically expected [34, 35]. The low value may be due to the relatively low incidence of MACE and the strong influence of competing events (non-MACE death), reflecting the complexity of the underlying biology and clinical reality. The calibration-in-the-large and calibration slopes showed perfect agreement between predicted and observed cumulative incidence, indicating the accuracy of absolute risk estimated by our model. The C statistic showed an acceptable ability to distinguish the order of event times among patients, and the decision curve analysis showed positive net benefit for employing our model for decision-making within a clinically reasonable range of thresholds (2–18%). The results (figure 3) showed that employing our model for decision-making provides a positive net benefit over the “treat-all” or “treat-none” strategies within a clinically reasonable range of thresholds, directly demonstrating its practical utility. Here, we only estimated the apparent net benefit values without performing an internal–external cross validation, which might be optimistic. Due to the large sample size, the bias is expected to be low and acceptable. The key utility of our model lies in risk stratification. When patients were categorised into low-, medium- and high-risk groups based on model predictions, the cumulative incidence curves demonstrated significantly different event rates between these strata. This ability to identify subgroups is directly relevant for guiding clinical decisions, such as intensifying therapy for high-risk patients.

### Strengths of the study

One of the strengths of this study lies in the comprehensive predictors evaluated. We included all important features reflecting the respiratory and cardiovascular status that are available and well recorded in real-world healthcare data as the candidate predictors. Among the final six predictors, apart from the CVD-specific disease history, severity of the COPD exacerbation and the mMRC dyspnoea scale were found to be important predictors. Severity of COPD exacerbation is a well-recognised risk factor of CVD [6], likely in relation to worsening of the factors that contribute to CVD (systemic inflammation, abnormal pulmonary gas exchange, gas trapping and lung hyperinflation). Additionally, cardiovascular mechanisms that can influence lung function (reduced myocardial contractility leading to pulmonary oedema, pulmonary hypertension and poor perfusion of systemic organs) also worsen during COPD exacerbation [2]. Our study showed the prediction effect of the mMRC scale for future MACE risk, emphasising the importance of normalised management and early intervention for improving not only respiratory status but also overall well-being.

Another strength is the use of large-scale linked primary and secondary healthcare datasets to reduce the risk of overfitting due to inadequate sample size. Moreover, we used the internal–external validation framework in this large, clustered healthcare dataset. Instead of a split-sample design that can result in a reduced opportunity for the model to learn, the current modelling strategy permitted robust assessment of model performance and heterogeneity across regions [22]. Although the random-effect of meta-analysis showed the heterogeneity of R^2^, C statistics and calibration-in-the-large across regions, the performance was very consistent given the big sample size. The results provide evidence to support the use of the CE-MACE model in UK primary and secondary care settings with confidence. The CE-MACE model was developed and validated in the context of the UK healthcare system. Future prospective external validations in other countries are required before it can be implemented internationally.

### Implications for clinicians

The CE-MACE model is valuable owing to its accessibility. For maximum generalisation and transparency of the CE-MACE model, we provide an online calculator and an easy-to-use score chart, together with a risk curve for a visual of prediction. The score chart was derived from the statistical model, retaining validity at the cost of negligible loss of performance. The sensitivity analysis showed that the CE-MACE model is valid for patients with any prior exacerbation history, expanding its applicability in the real-world healthcare context.

### Limitations of the study

Despite the mentioned strengths, our study has several limitations that should not be ignored. First, due to the data source, some potential predictors of underlying effects (*e.g.* genetic and blood biomarkers) were not available. Promising biomarkers for more accurate prediction have been investigated [36, 37], but the integration of biomarkers into prediction models for broad use remains a huge challenge. Second, definition and measurement of the predictors might be to some extent varied because the data were from a 15-year span and different GPs in England. However, most of the six predictors in the CE-MACE model are relatively objective and can be easily recorded in real-world settings with little bias. Moreover, we did not use modern statistical methods such as machine learning, although artificial intelligence has been highly developed in the current era. By using traditional statistical models and presenting the prediction tool in a visual way, we have simplified it for use in clinical practice. The model is hence very transparent for future independent external validation and transportability without technological barriers. Moreover, traditional statistical models show no disadvantages compared with modern models in many aspects [38]. The primary aim of this study was to develop a valid model rather than to make comparisons between different methods. Finally, missing values are a big, but inevitable, challenge in routinely collected healthcare data. We used the missing indicators for GOLD stage and mMRC that had high proportions of missingness, which is a recommended strategy for modelling study context with a MNAR assumption. We assume that missingness of these important predictors commonly occurs when deploying the model. By using the missing indicator, we properly developed the model and provide equal chances of risk evaluation for each patient. We acknowledge that the model is limited in that it can only be used if the five predictors (other than mMRC) are all complete when it is utilised.

### Conclusions

We have developed and validated the CE-MACE model for 1-year risk of MACE after exacerbation in patients with COPD, based on six routinely collected healthcare parameters, supporting application at low cost of data and technology. Implementation of the CE-MACE model could bridge the gap between consciousness and practice of cardiovascular risk for COPD and promote multidisciplinary integrated care.

## Data Availability

This study is based in part on data from the CPRD obtained under licence from the UK Medicines and Healthcare products Regulatory Agency. The data are provided by patients and collected by the NHS as part of their care and support. The interpretation and conclusions contained in this study are those of the authors alone. Data are available on request from the CPRD. Their provision requires the purchase of a licence, and this licence does not permit the authors to make them publicly available to all. This work used data from the version collected in December 2024 and has clearly specified the data selected within each Methods section. To allow identical data to be obtained by others, *via* the purchase of a licence, the code lists will be provided upon request. Licences are available from the CPRD (https://www.cprd.com): The Clinical Practice Research Datalink Group, The Medicines and Healthcare products Regulatory Agency, 10 South Colonnade, Canary Wharf, London, E14 4PU, UK.

## Supplementary material

**Please note:** supplementary material is not edited by the Editorial Office, and is uploaded as it has been supplied by the author. Supplementary material ERJ-02555-2025.Supplement CE-MACE Risk Model two-step tutorial and scoring chart ERJ-02555-2025.CE-MACE_Risk_Model
