# Supplementary material for: Developing and validating the CE-MACE model to predict 1-year major adverse cardiovascular events post-COPD exacerbation using routine healthcare data
Source: Eur Respir J. 2026 Jul 23;68(1):2502555. doi: 10.1183/13993003.02555-2025 (PMC13392454; doi:10.1183/13993003.02555-2025)
Supplement: Supplementary file 1 [file ERJ-02555-2025.Supplement.pdf]

Supplementary Material:

Developing and Validating the CE-MACE Model to Predict One-Year Major Adverse Cardiovascular Events Post COPD Exacerbation Using Routine Healthcare Data

Table S1. ICD-10 codes for determining severe COPD exacerbations. A severe exacerbation (hospitalizations for AECOPD in HES) was defined with the following ICD-10 codes and positions in the first episode of care:

1st position	2nd position
J44.0 - COPD with acute lower respiratory infection	Any code
J44.1 - COPD with acute exacerbation	Any code
J44.8 - other specified COPD	Any code
J44.9 - COPD unspecified	Any code
J43.9 - emphysema	Any code
J22 - unspecified acute lower respiratory infection	J44.0 or j44.1 or j44.8 or j44.9 or j43.9
R06.0-dyspnoea (shortness of breath)	J44.0 or j44.1 or j44.8 or j44.9 or j43.9
J96.0 - acute respiratory failure	J44.0 or j44.1 or j44.8 or j44.9 or j43.9
J96.1 - chronic respiratory failure	J44.0 or j44.1 or j44.8 or j44.9 or j43.9
J96.9 - respiratory failure	J44.0 or j44.1 or j44.8 or j44.9 or j43.9
J10.1 - influenza due to other identified influenza virus with other respiratory manifestations	J44.0 or j44.1 or j44.8 or j44.9 or j43.9

COPD, chronic obstructive pulmonary disease; AECOPD, acute exacerbation of COPD; HES, Hospital Episode Statistics; ICD, International Classification of Diseases.

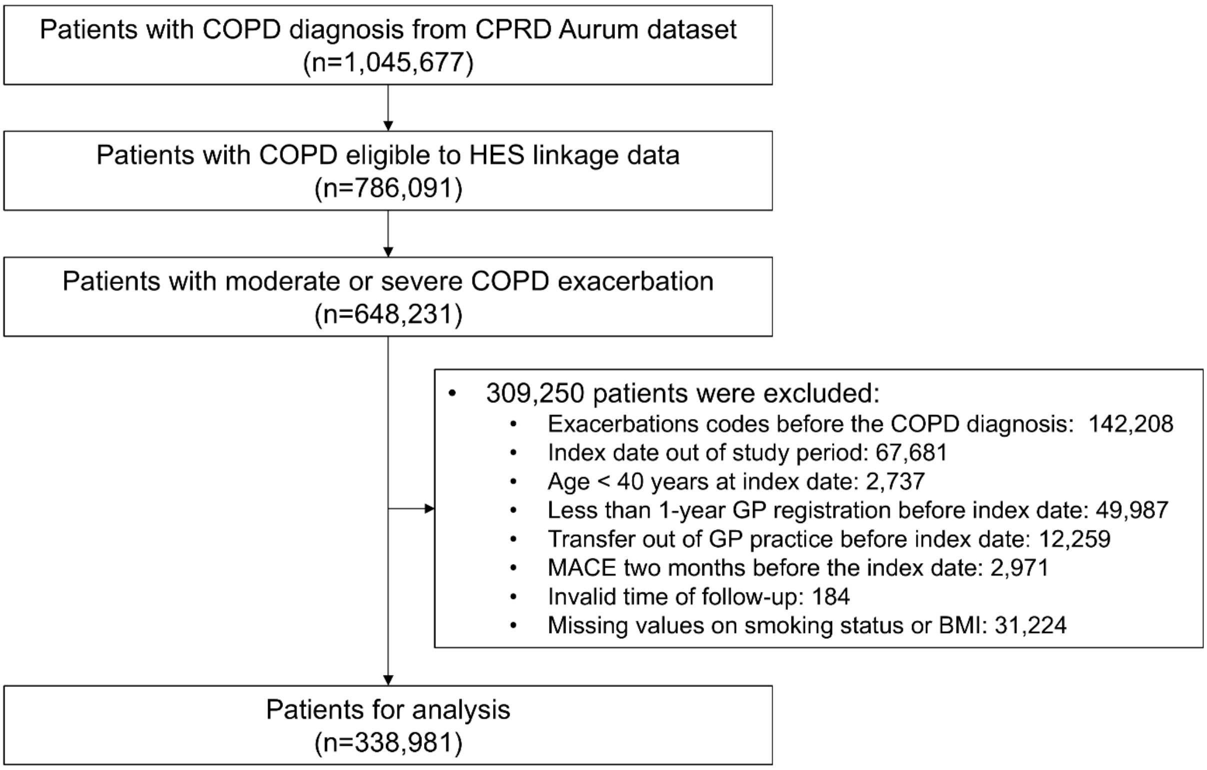

Figure S1. Flowchart for selecting study population. COPD, chronic obstructive pulmonary disease; CPRD, Clinical Practice Research Datalink; HES, Hospital Episode Statistics; GP, general practitioner; MACE, major adverse cardiovascular event; BMI body mass index.

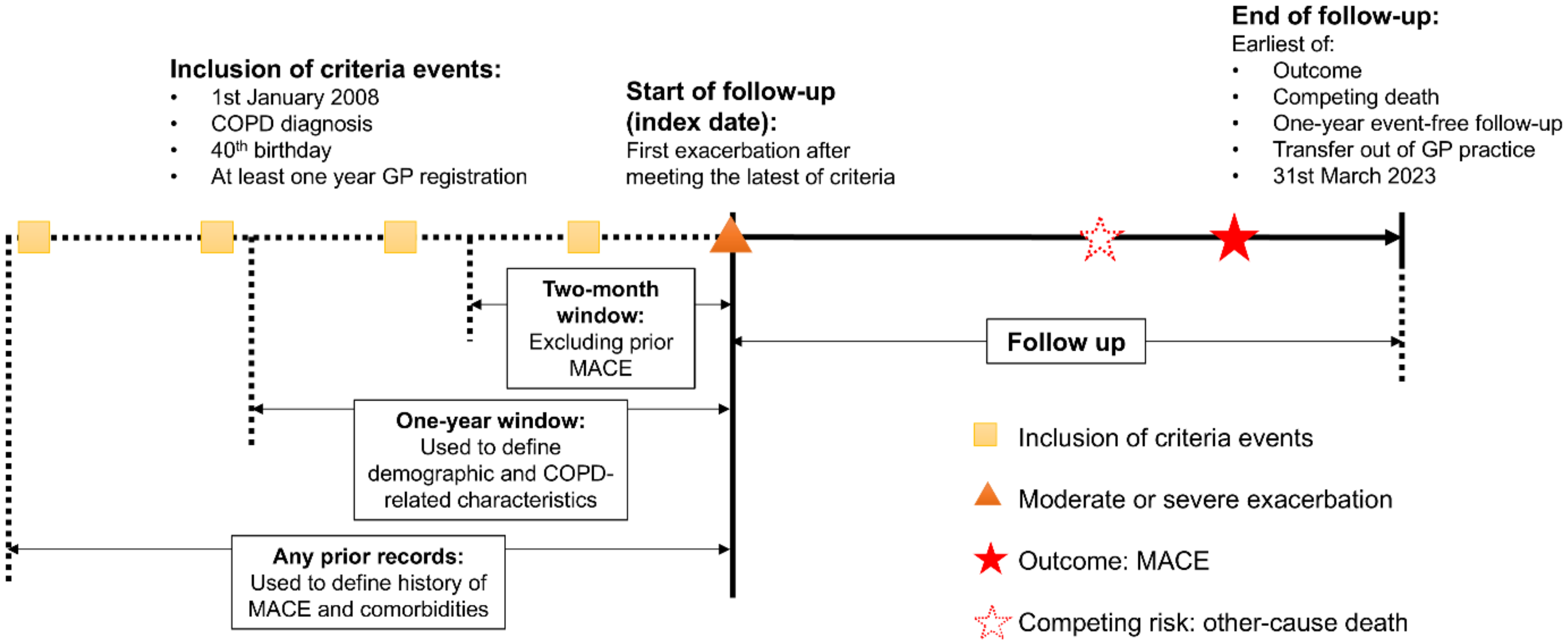

Figure S2. Study design for defining the study cohort. COPD, chronic obstructive pulmonary disease; GP, general practitioner; MACE, major adverse cardiovascular event

Table S2. Candidate predictors and the fraction of missing values

Category	Candidate predictors	Fraction of missing (%)	Note
Demographic characteristics	1. Age (years)	0	
	2. Sex	0	
COPD related characteristics and history	3. Smoking status	1.56	
	4. BMI (kg/m^2)	7.02	
	5. Severity of current exacerbation	0	
	6. COPD exacerbation in last 12 months	0	
	7. ICS use in last 12 months	0	
	8. FEV1/pred %	42.35	
	9. mMRC dyspnea scale	46.13	
Cardiovascular disease history	10. Prior history of ACS	0	Combined into one predictor.
	11. Prior history of arrhythmia	0	
	12. Prior history of heart failure	0	
	13. Prior history of stroke	0	
	14. Statins use in last 12 months	0	
Comorbidity	15. Hypertension	0	
	16. Diabetes	0	
	17. Asthma	0	
	18. Depression	0	
	19. Anxiety	0	
	20. GORD	0	
	21. LRTI	0	

COPD, chronic obstructive pulmonary disease; BMI, body mass index; ICS, inhaled corticosteroids; FEV1/pred %, percent predicted forced expiratory volume in one second; mMRC, modified British Medical Research Council; ACS, acute coronary syndrome; GORD, gastroesophageal reflux disease; LRTI, lower respiratory tract infection.

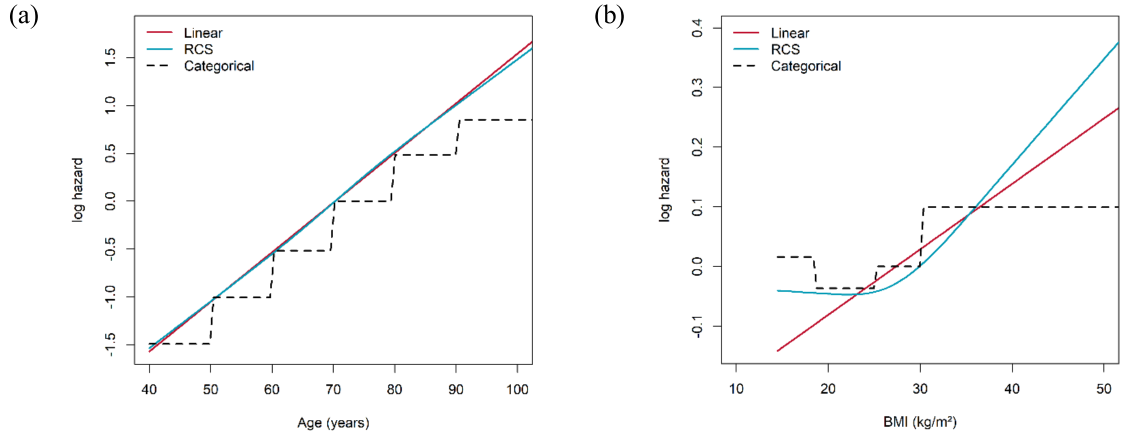

Figure S3. Linear, RCS, and categorical relationships between continuous candidate predictors with outcome. a) age, b) BMI. RCS, restricted cubic spline; BMI, body mass index.

Table S3. Comparison of linear, RCS, and categorical relationships between continuous candidate predictors with outcome

	df	Harrell's C statistic	Nagelkerke's R^2 (%)	LR statistic	AIC
Age (years)					
Linear	1	0.656	1.025	4812.8	392651.6
RCS	3	0.656	1.025	4816.2	392652.1
Categorical	5	0.646	0.959	4500.1	392808.5
BMI (kg/m^2)					
Linear	1	0.517	0.018	83	397381.4
RCS	3	0.517	0.021	99.1	397367.3
Categorical	3	0.515	0.011	49.6	397418.8

df, degree of freedom; LR, likehood ratio; AIC, Akaike information criterion; RCS, restricted cubic spline; BMI, body mass index.

Table S4. Estimated effect of MACE history to outcome

MACE history	Univariate coefficients	Multivariate coefficients	Score[a]	Model 1		Model 2		Model 3	
				Harrell's C statistic	Nagelkerke's R^2 (%)	Harrell's C statistic	Nagelkerke's R^2 (%)	Harrell's C statistic	Nagelkerke's R^2 (%)
ACS	1.103	0.627	3	0.705	2.147	0.704	2.146	0.677	1.763
Arrhythmia	1.300	0.880	4						
Heart failure	1.489	0.912	4						
Stroke	0.796	0.460	2						

[a] score was defined by the rounded relative magnitude of the coefficients; MACE, major adverse cardiovascular event; ACS, acute coronary syndrome. Model 1: four MACE history predictors were included in model independently; Model 2: scores was included in model as the integrated indicator of MACE history, scores ranging 0 to 13; Model 3: MACE history was included in model as yes or no (1 or 0).

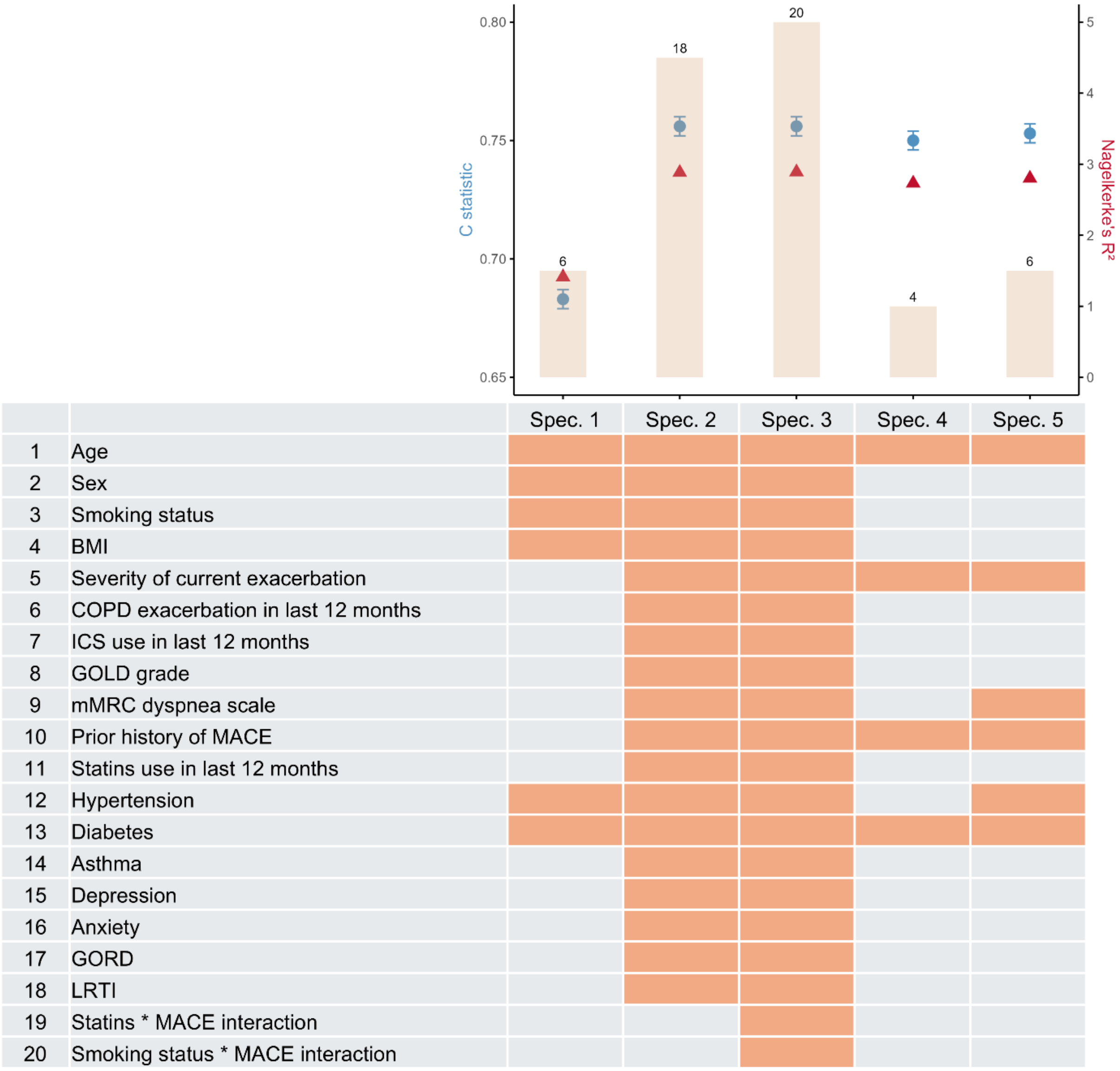

Figure S4: Comparison of different modelling specifications

Specification 1: Conventional cardiovascular disease risk factors.
Specification 2: Full model.
Specification 3: Specification 2 + interactions.
Specification 4: LASSO selected predictors.
Specification 5: Specification 4 + researcher selected predictors.
BMI, body mass index; COPD, chronic obstructive pulmonary disease; ICS, inhaled corticosteroids; GOLD, Global Initiative for Chronic Obstructive Lung Disease; mMRC, modified British Medical Research Council; MACE, major adverse cardiovascular event; GORD, gastroesophageal reflux disease; LRTI, lower respiratory tract infection. Red triangle is the Nagelkerke's R^2, blue dot and error bar is the Harrell's C statistics, and pink bar is the number of predictors for each model specification.

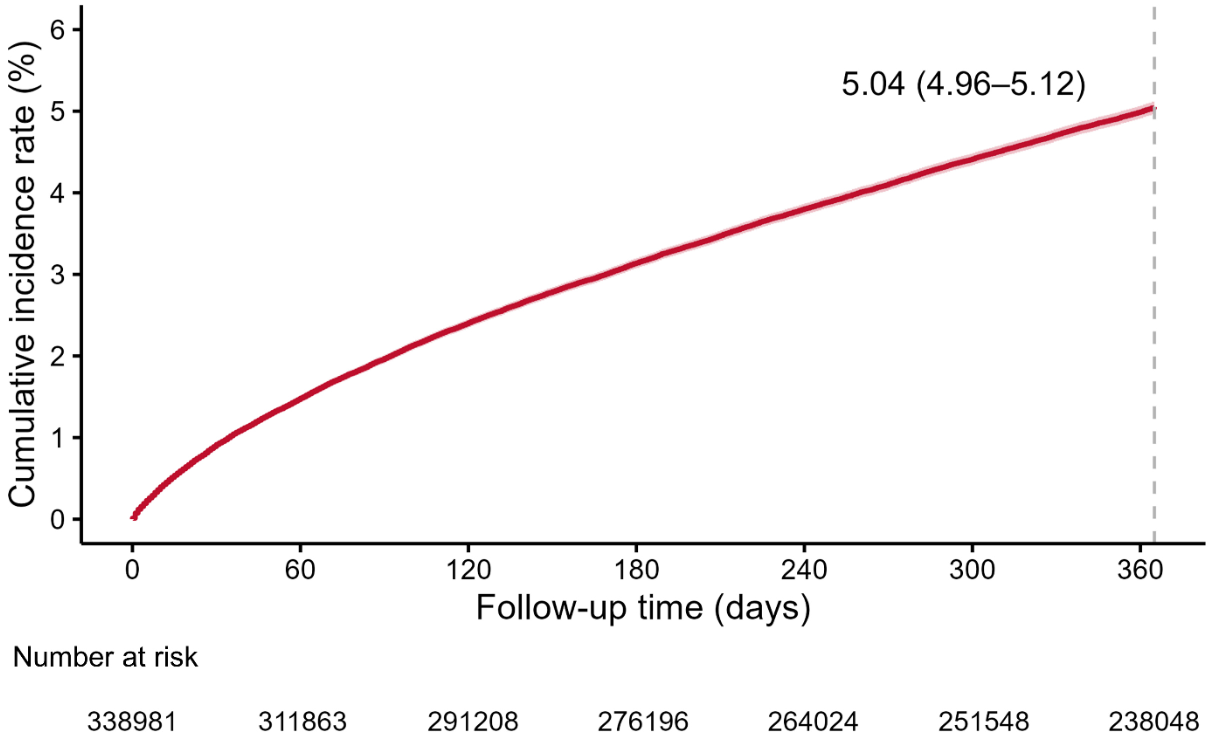

Figure S5. Cumulative incidence rate of MACE in the study cohorts. MACE, major adverse cardiovascular event.

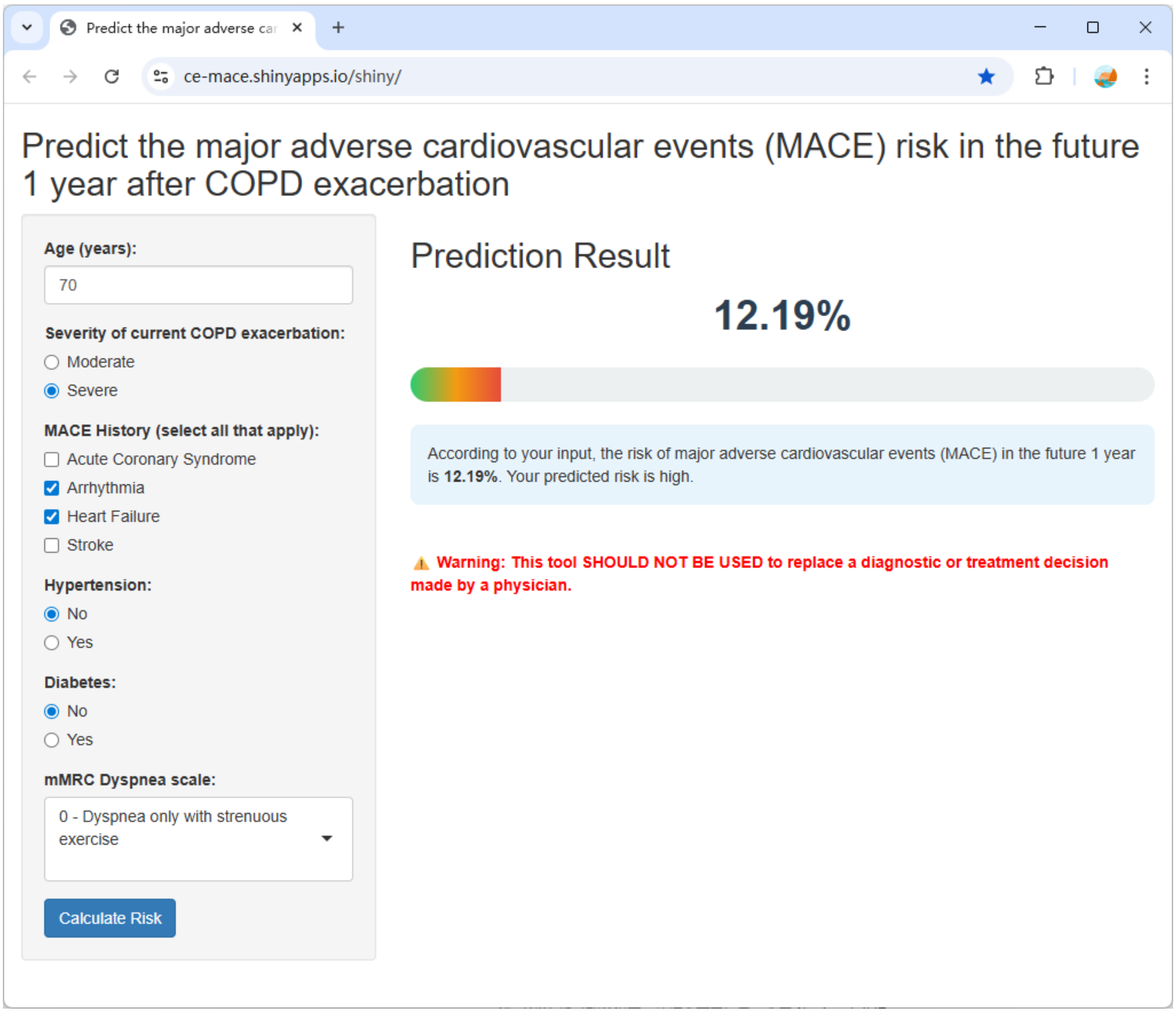

Figure S6. An example of prediction using the online calculator of the CE-MACE model at https://ce-mace.shinyapps.io/shiny/.

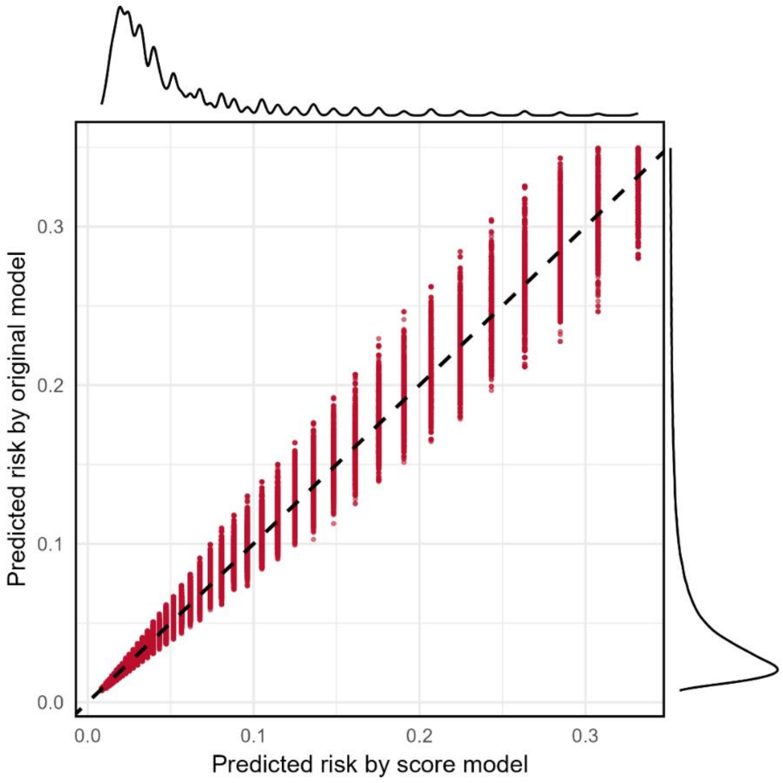

Figure S7. The agreement of the predicted risk by risk score and original model.

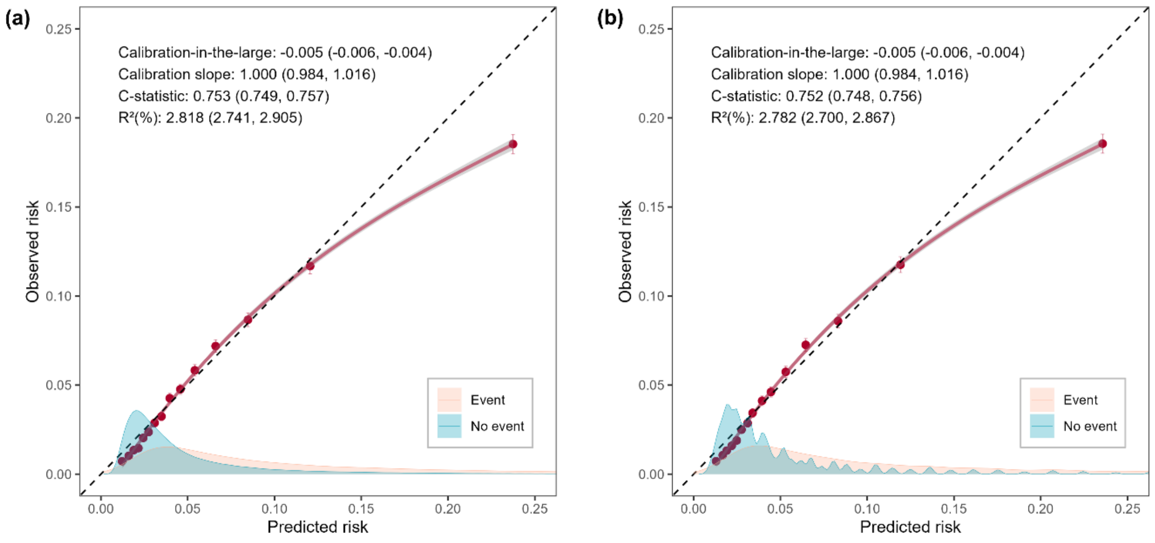

Figure S8. Apparent performance of CE-MACE model and distribution of predicted risk. (a) model in original format; (b) model in risk score format. Calibration curves and shaded intervals were smoothed by restricted cubic spline approach. Dots are grouped observations by percentile of predicted risk. Bars are 95% confidence intervals of observed cumulative incidence rates. The density curves in the bottom show the distributions of predictions in those with and without event.

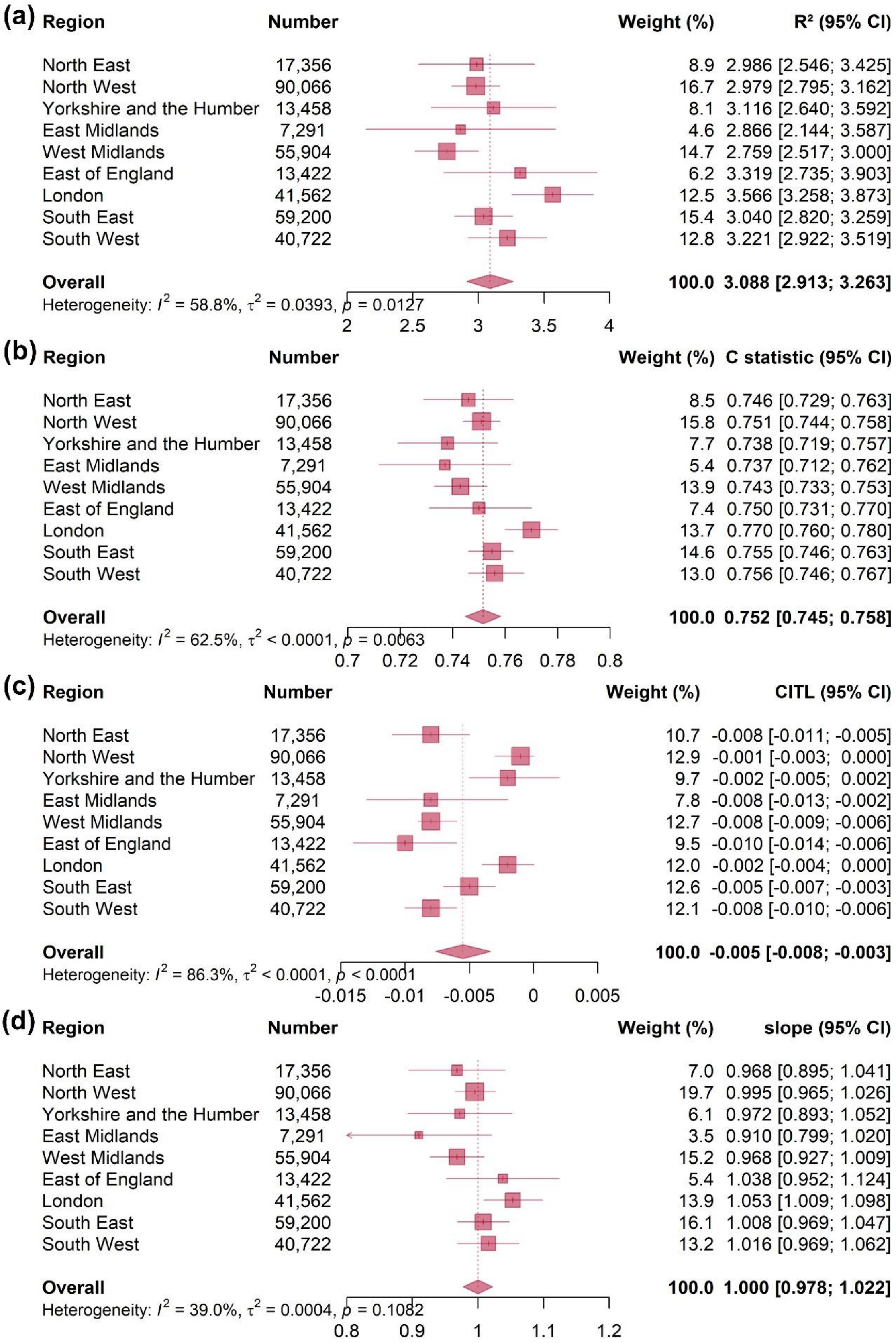

Figure S9. Results from internal-external cross validation of risk score model performance metrics. Plots display region level performance metric estimates and 95% confidence intervals, and an overall pooled estimate obtained using random effects meta-analysis. (a) Nagelkerke's R^2; (b) Harrell's C statistics; (c) calibration-in-the-large; (d) calibration slope.

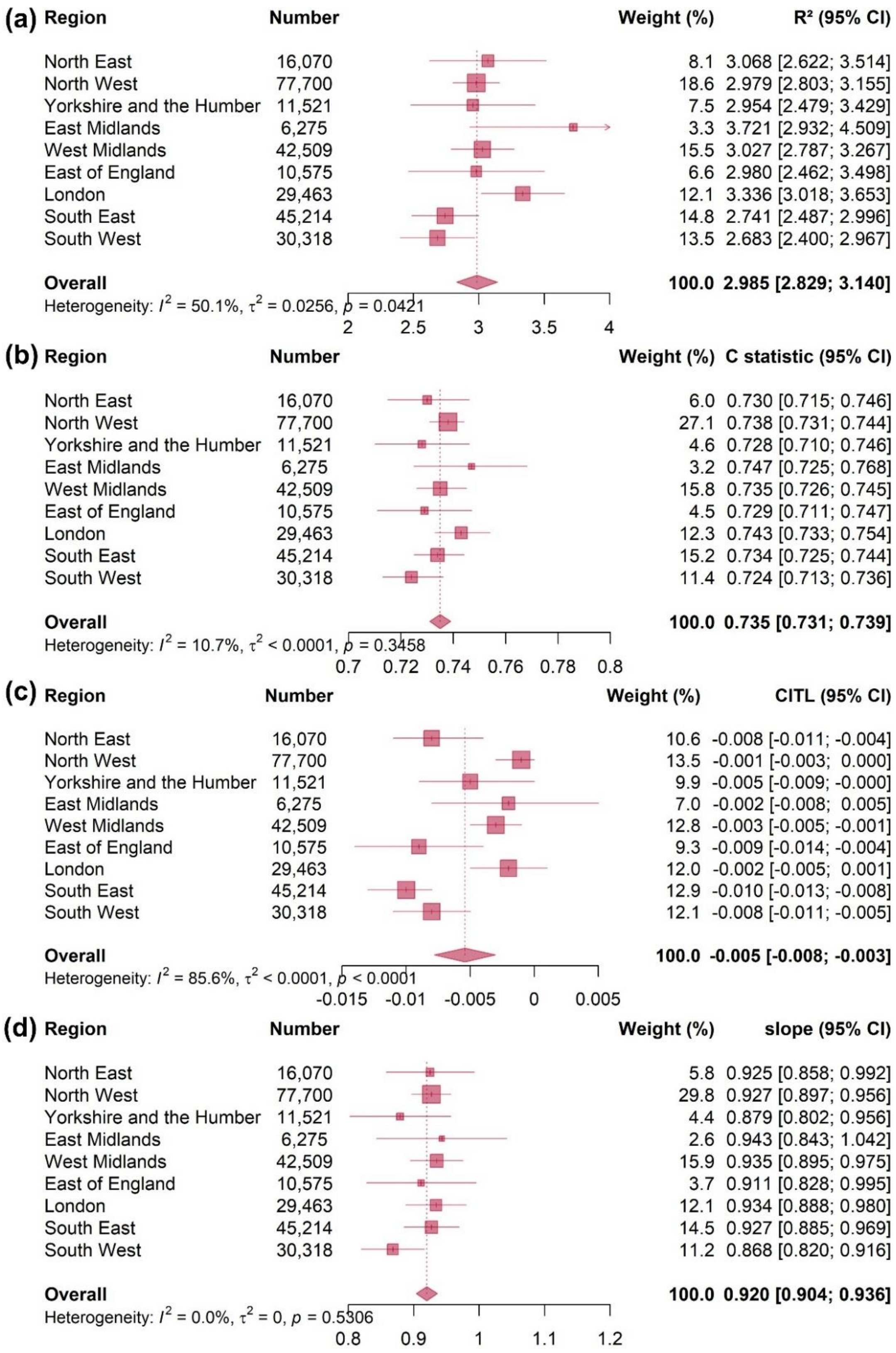

Figure S10. Sensitivity analysis results of internal-external cross validation of CE-MACE model performance metrics using subsequent exacerbations as analysis units. Plots display region level performance metric estimates and 95% confidence intervals, and an overall pooled estimate obtained using random effects meta-analysis. (a) Nagelkerke's R^2; (b) Harrell's C statistics; (c) calibration-in-the-large; (d) calibration slope.

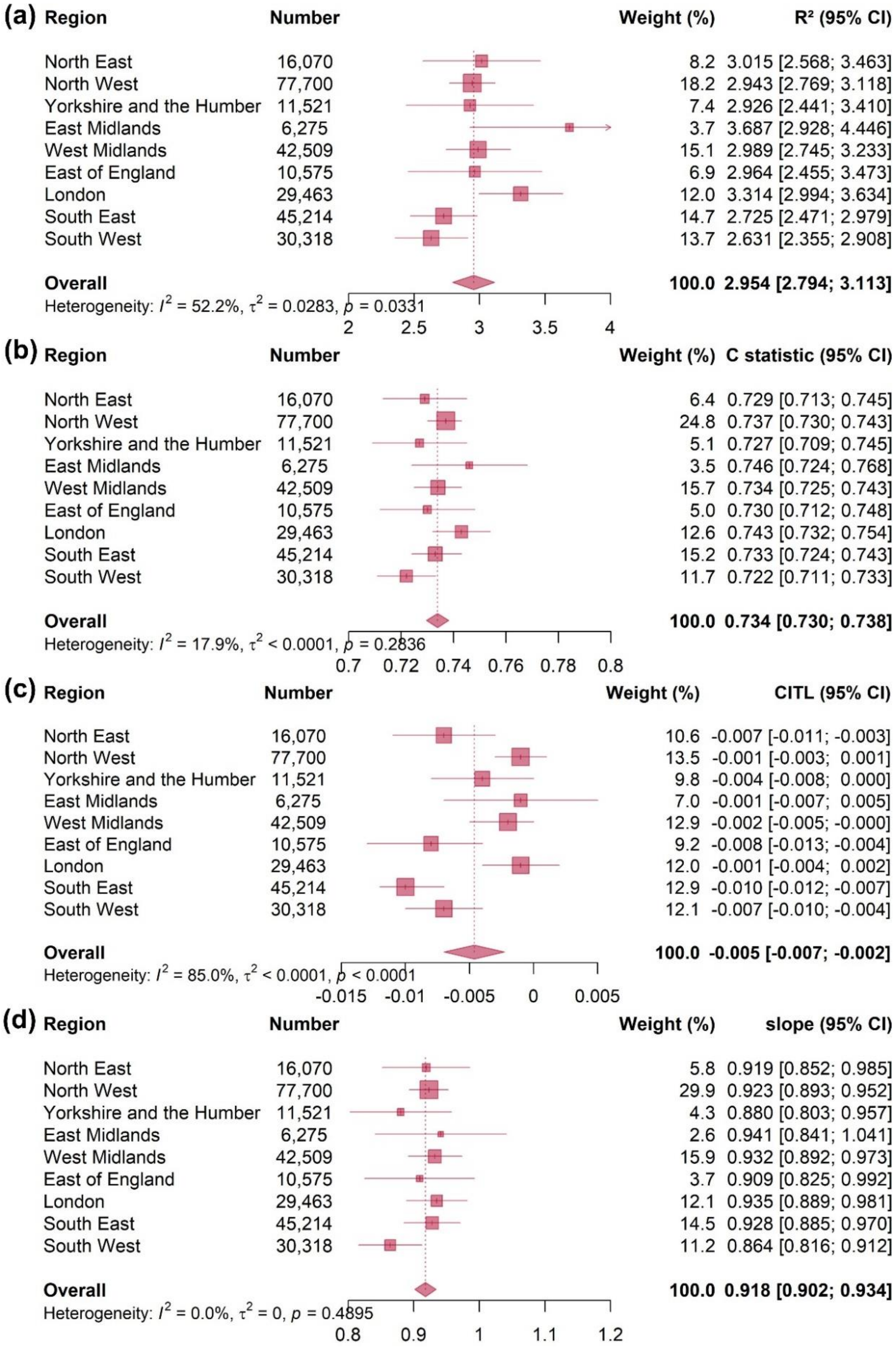

Figure S11. Sensitivity analysis results of internal-external cross validation of risk score performance metrics using subsequent exacerbations as analysis units. Plots display region level performance metric estimates and 95% confidence intervals, and an overall pooled estimate obtained using random effects meta-analysis. (a) Nagelkerke's R^2; (b) Harrell's C statistics; (c) calibration-in-the-large; (d) calibration slope.

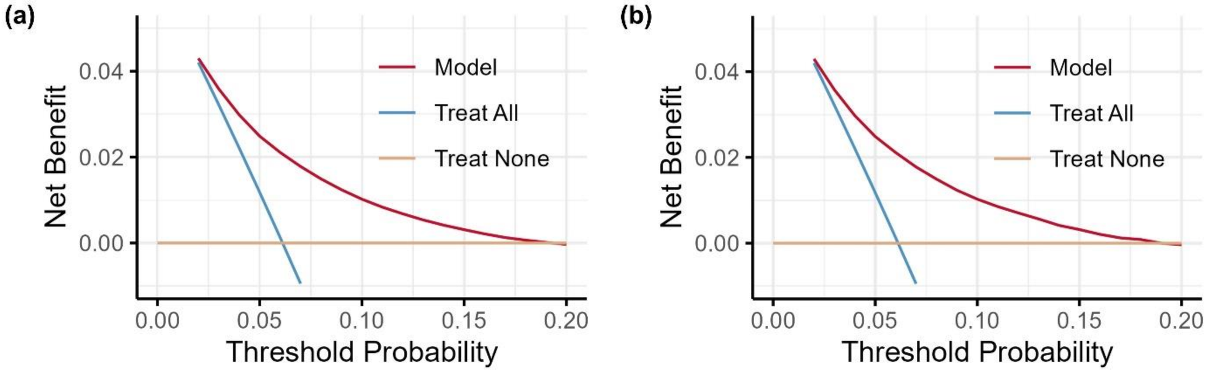

Figure S12. Sensitivity analysis results of decision curves analysis using subsequent exacerbations as analysis units. (a) model in original format; (b) model in risk score format
