# Supplementary material for: Developing and validating the CE-MACE model to predict 1-year major adverse cardiovascular events post-COPD exacerbation using routine healthcare data
Source: Eur Respir J. 2026 Jul 23;68(1):2502555. doi: 10.1183/13993003.02555-2025 (PMC13392454; doi:10.1183/13993003.02555-2025)
Supplement: Supplementary file 2 [file ERJ-02555-2025.CE-MACE_Risk_Model.pdf]

CE-MACE Risk Model

The CE-MACE model is used to assess the risk of major adverse cardiovascular events after a moderate or severe COPD exacerbation.

HOW TO USE CE-MACE RISK MODEL?

STEP 1: Calculate the Risk Score

**Terms**	**Categories**	**Score**	**Score assigned**
Age (years)	40-44	12	
	45-54	15	
	55-64	18	
	65-74	21	
	75-84	24	
	85-94	27	
	95-	30	
Severity of current COPD exacerbation	Moderate	0	
	Severe	5	
MACE history	Acute coronary syndrome	6	
	Arrhythmia	8	
	Heart failure	8	
	Stroke	4	
Hypertension	No	0	
	Yes	2	
Diabetes	No	0	
	Yes	3	
mMRC dyspnea scale	0-Dyspnea only with strenuous exercise	0	
	1-Dyspnea when hurrying or walking up a slight hill	1	
	2-Walks slower than peers due to dyspnea	3	
	3-Stops for breath after walking 100 yards	4	
	4-Too dyspneic to leave house	5	
	Not available/Missing	3	
SUM SCORE			

NOTE:

(a) In the context of the UK healthcare system, a moderate COPD exacerbation is that managed in primary care, a severe COPD exacerbation is that managed in secondary care.

(b) Histories of MACE, hypertension, and diabetes are any such histories before current exacerbation.

(c) mMRC dyspnea scale is the latest assessment one-year before current exacerbation.

STEP 2: Find the Predicted Risk from the Curve

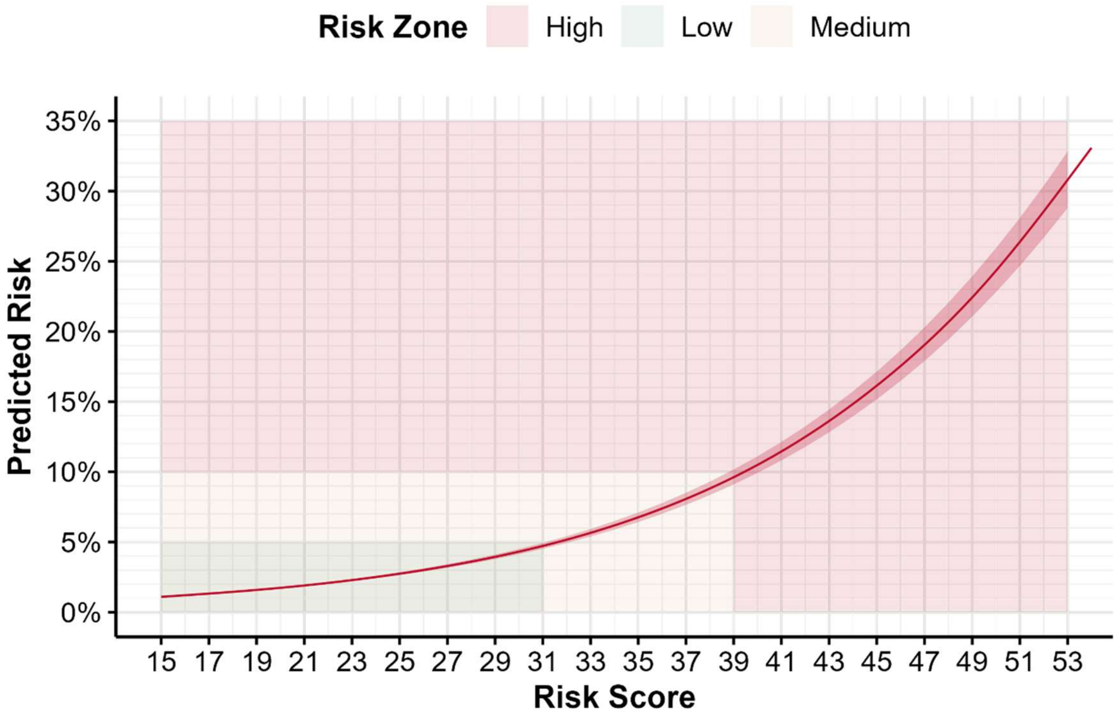

NOTE:

(a) Risk score in x-axis is the sum scores calculated from STEP 1.

(b) Predicted risk in y-axis means the one-year predicted risk of fatal or non-fatal MACE (including acute coronary syndrome, arrhythmia, heart failure, or stroke).

(c) High-risk group: predicted risk ≥10%; medium-risk group: predicted risk 5% – <10%; low-risk group: predicted risk <5%.
